# Evidence of maternal and paternal age effects on speed in thoroughbred racehorses

**DOI:** 10.1098/rsos.220691

**Published:** 2022-10-05

**Authors:** Patrick Sharman, Andrew J. Young, Alastair J. Wilson

**Affiliations:** Centre for Ecology and Conservation, University of Exeter, Penryn Campus, Penryn, Cornwall TR10 9FE, UK

**Keywords:** parental ageing, senescence, performance, within-individual centring, selective disappearance

## Abstract

Effects of parental age on offspring viability have been reported in a wide range of species. However, to what extent parental age influences offspring traits beyond viability remains unclear. Moreover, previous research has primarily focused on maternal age effects. The purpose of this study was to test for paternal and maternal age effects on offspring speed in thoroughbred racehorses. We analysed over 900 000 race performances by over 100 000 horses on British racecourses between 1996 and 2019. With knowledge of the age of all 41 107 dams and 2 887 sires at offspring conception, we jointly modelled maternal and paternal age effects using a ‘within-individual centring’ approach. Within-parents, we identified a significant effect of maternal age on offspring speed of −0.017 yards s^−1^ yr^−1^ and a corresponding paternal age effect of −0.011 yards s^−1^ yr^−1^. Although maternal age effects were stronger (more negative), the existence and magnitude of paternal effects is particularly noteworthy, given thoroughbred sires have no involvement in parental care. Our results also suggest that the selective disappearance of both sires and dams is ongoing. These findings could potentially be used to optimize thoroughbred racehorse breeding decisions, and more generally, add to the increasing body of evidence that both maternal and paternal age affect a range of offspring characteristics.

## 1. Introduction

Parental effects occur when the parental phenotype directly influences that of the offspring in ways over and above direct genetic inheritance. They have long been recognized as an important source of variation in offspring traits and can have major consequences for responses to selection. These consequences, which arise whether selection on offspring traits is artificial [1] or natural [2], stem from two well-recognized issues. First, if parental effects are present but unrecognized, this can bias estimates of additive genetic variance, typically leading to inflated expectations of response to selection. Second, if parental effects stem from among-parent differences in traits that are themselves heritable, then co-evolutionary dynamics can arise [3]. Less widely considered, at least in an applied animal breeding context, is that parental effects on offspring traits are potentially labile within parents, changing across reproductive episodes as a function of parental ‘state’ (see [4,5] for ecological examples). Here, we focus on parental age at breeding (hereafter parental age), an aspect of state that has been shown to influence offspring in a number of long-lived iteroparous species. We ask whether, and to what extent, parental ageing has consequences for the expression of a commercially important livestock trait under selection in offspring: running speed in thoroughbred racehorses.

Although effects of parental age have been documented across many taxa [6], our understanding of their prevalence and magnitude is limited in two important respects. First, the extent to which different aspects of offspring phenotype are impacted remains unclear because the great majority of studies have only considered offspring survival. For example, in an early human study, which focused on the descendants of early American settler William Hyde, Bell [7] reported that children of older parents had shorter lifespans. Reduced lifespan and/or a decline in early life survival, generally interpreted as evidence of senescent decline in parental performance, has since been observed in plants [8], invertebrates [9–12] and both captive and wild vertebrates [13,14]. By contrast, we generally know little about how parental age influences other aspects of offspring phenotype (but see [8,15,16] for studies on reproductive success and weight).

A second gap in our knowledge relates to the importance—or otherwise—of paternal age effects. This is because the vast majority of research on parental age effects has focused on maternal age only (but see [7]). The focus on mothers is partly pragmatic since, especially in unmanaged populations, paternity is unknown while maternal relationships can more often be inferred through observation. More generally, however, a focus on mothers reflects the fact that provision of parental care is the most obvious route to parental effects [17], and females provide the bulk of parental care across most animal taxa. This is particularly true if care is broadly defined so as to include prenatal investment (e.g. egg and/or fetal provisioning) as well as post-natal care (e.g. milk provisioning, feeding and defence). Senescent declines in maternal care are thus a plausible mechanism by which negative effects of maternal age on offspring viability might arise. However, additional mechanisms are possible. For instance, in mammals, the production of primary oocytes ends before birth, and there is now some evidence that the female germline can exhibit degenerative change with advancing age [18]. Epigenetic effects on offspring traits (mediated by, for example, DNA methylation, histone modification and transfer of non-coding RNAs) can also be sensitive to maternal environment [19,20], including age. For example, increased maternal age has been associated with altered DNA methylation patterns in offspring in humans [21].

In reality, not all these possible mechanistic pathways are specific to mothers; fathers provide at least some post-natal parental care in many species [22]; sperm of older men have less stable DNA (associated with increased rates of genetic disease in the resulting offspring [23]); and epigenetic signals are transmitted in ejaculates [24,25]. It is therefore feasible that paternal age could contribute more to offspring variation than is widely recognized at present. Several recent studies support this view. In wandering albatrosses, which actually exhibit primarily paternal post-hatching care, paternal age negatively affects juvenile survival while maternal age does not [13]. Paternal and maternal age were found to have negative effects of similar magnitude on offspring lifespan in neriid flies [12], while paternal age effects have also been reported in *Drosophila* [26], butterflies [27], sparrows [28], common terns [29] and mice [14].

In this study, we seek to characterize maternal and paternal age effects on speed in thoroughbred racehorses. Parental age effects on production and performance traits have received little scrutiny in livestock generally, or horses specifically (but for maternal age effects on milk production in dairy cattle see [30–32]). This is surprising given that (i) parental age at breeding can (usually) be controlled and (ii) the commercial value of livestock breeding industries can be very high. For example, horses selling for a total of £247 million went through the two premier thoroughbred auction houses in England (Tattersalls and Doncaster Bloodstock Sales) in 2012 alone [33], and successful horses (especially stallions) regularly earn far more over their breeding careers than they do racing. Moreover, breeding careers can be long—successful horses may retire from racing at 2 or 3 years old but breed well into their 20s—highlighting the potentially economic significance of parental age effects on offspring performance.

Although published estimates of parental age effects in horses are limited, maternal age-related declines in foal birth weight [34–36] and placental viability [36] have been reported. There are few studies on race performance, but those that do exist point to an initial increase in offspring performance with maternal age followed by a steady decline [37–39]. For example, in a sample of 100 dams with offspring running in Great Britain between 1947 and 1990, an increase in offspring Timeform rating (a widely used racehorse performance metric in Great Britain and Ireland) to maternal age 9 was detected, after which there was a decline [37]. Analysed by maternal parity number (i.e. the number of times a mare has given birth) rather than age, average offspring Timeform ratings increased to the fourth parity before declining [37]. In practice, parity and age are strongly correlated making their effects difficult to disentangle. A larger study of horses in the USA (including 18 931 offspring from 1641 mares) found that offspring of parities 2–5 are most likely to win a high-level race [38]. There are, to our knowledge, no published studies that explicitly estimate paternal age effects on racehorse performance. However, it was reported in 1959 that the majority of winners of prestigious races in Poland and the (then) Soviet Union were sired by stallions that were early in their breeding careers [40]. More recently, evidence of declining success of leading stallions at older ages has been recognized by some within the industry [41,42].

The aim of the present study is to test for, and estimate the magnitude of, parental age effects on offspring speed in thoroughbred racehorses. We do this using a much larger dataset than previous investigations, analysing 906 027 finishing times by 101 257 (offspring) horses in 88 385 races run in Great Britain between 1996 and 2019. Knowledge of parental identities of all offspring and the age of all 41 107 dams and 2887 sires at offspring conception allows us to jointly model maternal and paternal age effects. We define these parental age effects as being within-individual (parent) changes in offspring speed with (parental) age and adopt a ‘within-individual centring approach’ [43] widely used in ecological studies. This allows us to isolate true effects of within-parent changes in age from the potentially confounding effects of differences in age among-parents, which could arise through alternative processes such as selective appearance and/or disappearance. For instance, if lower quality parents are retired from breeding early, then their ‘selective disappearance’ means the population level relationship between observed parental age and offspring performance will underestimate the rate of (within-parent) senescence [44].

We predict, based on the limited literature available, that maternal age effects will be present, with a trend towards lower offspring speed at high maternal ages. Our expectation, is that paternal age effects will, if detectable, be of a lesser magnitude than maternal age effects. This follows from the fact that, in thoroughbred breeding, dams provide parental care until the foal is around six months of age. By contrast sires do not contribute to care and provide only ejaculate. Nonetheless, while there is no prior evidence of ejaculate-mediated paternal effects in horses, they are now increasingly being detected across taxa [24]. In this context, and set within the commercial drivers of the horse breeding industry, detection of paternal age effects would have important fundamental and applied implications.

## 2. Methods

### 2.1. Data source

The dataset used comprised 906 027 finishing time records by 101 257 different (offspring) horses running across 88 385 individual races in Great Britain between 1996 and 2019. This only included races run ‘on the flat’ (as opposed to races involving jumps) and on turf (i.e. grass, not all-weather track surfaces). Finishing times were converted to running speed to provide a more intuitive performance trait. For each horse (offspring) contributing race records, maternal and paternal identities were known, as was the year of birth for all horses (offspring and their parents). This allowed calculation of maternal and paternal age (at breeding) for each individual offspring. In total 41 107 dams and 2887 sires are represented by offspring records. Maternal age ranged from 2 to 26 years, while paternal age ranged from 2 to 29 years. A summary of the maternal and paternal data is presented in electronic supplementary material, figure S1. Note parental age is defined for offspring *i* with parent *j* as *j*'s age at the breeding event that produced *i*. Thus, parental age is constant for an offspring individual (*i*) but varies within a parent (*j*) across its offspring. Performance records and pedigree data were supplied under licence for the current use by TBGenerations Limited, along with the horse- and race-level covariates used in our statistical models (see below).

### 2.2. Analysis

Data were analysed using a series of four linear mixed effect models of speed fitted in ASReml (v. 3) [45]. The four models differ in the way we specify the hypothesized effects of parental age (shown below) but otherwise included a common set of fixed and random effects. The former were to control for race- and individual-level covariates already known to influence speed but not relevant to the current hypotheses. In practice, we largely replicated the fixed effect structure used in previously published work to test for temporal trends in thoroughbred performance (speed) over time [46]. We refer the interested reader to that work for more detail on the choice of covariates. However, in brief, we included: offspring *age* at performance (in years), offspring *sex*, *racecourse*, *timing method* (hand-timed or automatic) and *year* of race as fixed factors. This was to condition speed on these effects, without assuming any functional form of, for example, trends over time (year of race) or offspring age. We also included linear and quadratic effects of race *distance* (in yards, the standard unit of measurement in British horseracing where 1 yard = 0.914 m), ‘*going*’ (a continuous measure of how soft the ground is) and the number of runners in the race (*no.runners*). Race *distance* was rescaled so that 0 corresponds to 1097 yards, the shortest race distance represented in the data, and we mean centred *going* (i.e. 0 corresponds to ground of average softness) and *no.runners* (i.e. 0 corresponds to average number of runners). This was simply to ease biological interpretation of model parameter estimates. Finally, based on previous analysis supporting their contribution to variation in speed [46], we also included interactions of *year* by *distance*, *year* by *distance^2^*, *distance* by *going* and *distance* by *no.runners.* Note for the pragmatic reason of avoiding very large numbers of model parameters, we elected to treat *year* as a continuous variable in interaction terms (but as a factor for the main effect) and rescaled so 0 corresponds to 1996 (the first year with performance records in the dataset). We then included random effects of horse (offspring) identity, dam identity and sire identity to account for non-independence of repeated observations records within these grouping factors. We make the standard assumptions that random effects (and residual errors) are normally distributed with means of zero and variances to be estimated.

Four linear mixed models were then sequentially fitted in which we used additional fixed effects to test for influences of parental age while conditioning on primiparity of mothers (for reasons highlighted below). In Model 1, the observed *speed* of offspring individual (*horse*) *i*, with mother (*dam*) *j*, and father (*sire*) *k* in race *R* was specified as follows:

Model 1 $${\mathrm{Speed}}_{iR}∼\mu_{F}+{\mathrm{primiparity}}_{ij}+maternal\;ageF_{ij}+paternal\;ageF_{ik}+{\mathrm{horse}}_{i}+{\mathrm{dam}}_{j}+{\mathrm{sire}}_{K}$$

where *µ_F_* is the fixed effect mean conditional on all fixed effects described above; *horse*, *dam* and *sire* are random identity effects; *primiparity_ij_* is a fixed factor denoting whether *i* is the offspring of *j*'s first parity or not; and *maternal ageF_ij_* and *paternal ageF_ik_* are the parental ages (i.e. ages of *j* and *k* when breeding to produce *i* occurred) treated as multi-level factors (denoted by *F*). This model (Model 1) was used to first characterize the overall form of any parental age-related changes in population mean offspring speed. The factorial specifications of maternal and paternal age avoid assuming any particular functional form of parental age effects. In practice, there were very few offspring produced by 2-year-old sires (*n* = 2) and late paternal age categories also had low numerical representation (paternal ages 27, 28 and 29 represented by *n* = 35, 10 and 3 offspring, respectively). We therefore collapsed factor levels at these paternal ages for Model 1, pooling into categories of paternal age ≤ 3 (*n* = 445) and ≥ 26 (*n* = 175). We similarly created two pooled age factor levels for dams, corresponding to maternal age ≤ 3 (*n* = 1786) and maternal age ≥ 22 (*n* = 232). Previous studies have suggested qualitative differences between first-born foals and offspring of later parities; foals of primiparous dams tend to be lighter [34,36] and perform less well [37,38]. Since the intended focus of our study is on parental age (rather than parity), we elected to condition estimates of parental age effects on primiparity rather than parity number. Primiparity occurs—by definition—at the earliest age of reproduction within each dam, but the age of first breeding differs among-dams in the dataset, allowing these two processes to be statistically separated. By contrast, we expect parity number, if treated as a continuous covariate to be effectively collinear with maternal age. Therefore, we did not include this as an additional continuous covariate.

While Model 1 makes no assumption about the functional form of parental age effects, inferring within-individual (parent) change from a population-level analysis is vulnerable to bias from selective (dis)appearance. This can be dealt with by using within-subject centring [43] to more robustly partition within-individual and among-individual sources of variance (e.g. [47]). Since this approach requires treating parental ages as continuous predictors, we first replaced the factorial treatments of parental age used in Model 1 with linear covariates. This was simply to assess whether this was sufficient to capture the major patterns in population mean.

Model 2 $${\mathrm{Speed}}_{iR}∼\mu_{F}+{\mathrm{primiparity}}_{ij}+maternal\;age_{ij}+paternal\;age_{ik}+{\mathrm{horse}}_{i}+{\mathrm{dam}}_{j}+{\mathrm{sire}}_{K}$$

We then applied within-subject centring to partition the linear effects of dam *j*'s age on offspring performance, into a term reflecting the average age of *j* (across all its offspring in the dataset; $\;\bar{\mathrm{maternal}\;\mathrm{ag}e_{j}}$) and a deviation from this, specific to the offspring horse *i* (damΔ*_ij_*) where $\mathrm{maternal}\_{\mathrm{age}}_{ij}=\bar{\mathrm{maternal}\;\mathrm{ag}e_{j}}+\mathrm{dam}\Delta_{ij}.\;$Paternal age is then treated identically, such that $\mathrm{paternal}\_\mathrm{ag}e_{ik}=\bar{\mathrm{paternal}\;\mathrm{ag}e_{k}}\;+\mathrm{sire}\Delta_{ik}.$

Model 3 $$\begin{array}{rl} & {\mathrm{Speed}}_{iR}∼\mu_{F}+{\mathrm{primiparity}}_{ij}+\bar{maternal\;age_{j}}+dam\Delta_{ij}+\bar{paternal\;age_{k}}+sire\Delta_{ik}\\ & \;+{\mathrm{horse}}_{i}+{\mathrm{dam}}_{j}+{\mathrm{sire}}_{K} \end{array}$$

Note that Model 3 is a re-parametrization of Model 2 so will provide an identical fit to the data. However, under the Model 3 formulation, the regression coefficients associated with $\mathrm{dam}\Delta_{ij}$ and $\mathrm{sire}\Delta_{ik}$ provide estimates of the average (linear) dependence of offspring performance on age change within each parent. These estimates are robust to any age-related selective (dis)appearance of parents, since the latter process results in among-mother and/or among-father differences in average age at which offspring are born, which are separately modelled by the inclusion of $\bar{\mathrm{maternal}\;\mathrm{ag}e_{j}}$ and $\bar{\mathrm{paternal}\;\mathrm{ag}e_{k}}$**_._** Under this model, selective disappearance will result in (for the maternal case) a difference between the regression coefficients associated with $\mathrm{dam}\Delta_{ij}$ and $\bar{\mathrm{maternal}\;\mathrm{ag}e_{j}}$. Although this can be assessed by qualitative comparison of these coefficients as estimated under Model 3, it has previously been noted [43], that a statistical test for selective disappearance is easily carried out using one further model re-parametrization where

Model 4 SpeediR∼μF+primiparityij+maternal ageij+paternal ageik+maternal agej¯+paternal agek¯+ horsei+damj+sireK

Here, with all terms as already defined above, the regression coefficients for $\bar{\mathrm{maternal}\;\mathrm{ag}e_{j}}$ and $\bar{\mathrm{paternal}\;\mathrm{ag}e_{k}}$ are now estimated conditional on the observed parental age (maternal age*_ij_*, paternal age*_ik_*). This means they actually provide direct estimates of (and statistical inference on) the magnitude of selective disappearance (see [43] for further explanation).

Statistical significance of fixed effects was assessed using conditional *F*-tests implemented in ASReml (v. 3) with the denominator degrees of freedom estimated using the numerical derivatives method of Kenward & Roger [48]. Inference on random effects is not directly relevant to our objectives, but for completeness, we also tested each random effect (using the Model 3 formulation of fixed effects) by likelihood comparison of the full model to reduced versions in which the corresponding random effect was omitted. For testing each random effect variance component in this way, we assume that twice the difference in model log-likelihoods is distributed as a 50 : 50 mix of *χ*^2^_0_ and *χ*^2^_1_ distributions [49] which we denote as *χ*^2^_0,1_. For purposes of reporting standardized random effect sizes, we also estimated the intra-class correlation for each random effect as the ratio of the corresponding variance to total variance conditional on fixed effects (determined as the sum of variance component estimates from Model 3).

## 3. Results

**Figure 1.**
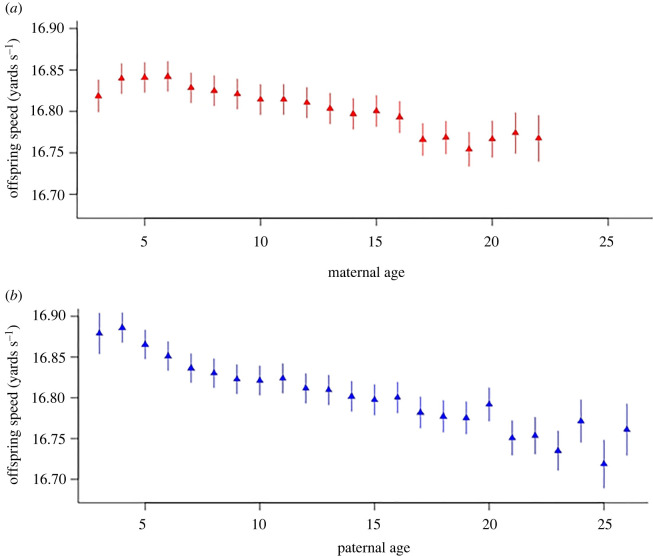
Predicted horse (offspring) speed by (*a*) maternal and (*b*) paternal age in years. Predictions are based on Model 1 treating parental ages as factors and averaging across all other fixed effects. Note some parental age factor levels were collapsed (see main text) such that the youngest age class is 3 years (maternal and paternal) while the highest is 22 years (maternal) and 26 years (paternal). Error bars denote ± 1 s.e.

Here we focus on results pertaining to the hypothesized parental age effects but note that tables including all estimated model parameters are provided in the electronic supplementary material, table S1. Under Model 1, we found strong statistical support for differences in average offspring speed across factor levels of both maternal age (*F*_19,78,958.9_ = 16.96, *p* = <0.001) and paternal age (*F*_23,73,713.3_ = 11.81, *p* = <0.001). Visualizing effect size estimates for each level of the factor shows that as maternal age increases, average offspring speed declines (figure 1*a*), and that the same pattern is seen with respect to paternal age (figure 1*b*). We note that these patterns are imperfect, since for both dams and sires estimated parental age effects actually show an initial increase—to age 6 for dams and 4 for sires (figure 1)—before declining (approximately) linearly. However, given our goal of summarizing parental age effects across the full range of ages represented in the population, it is reasonable (in our judgement) to approximating these patterns with linear (i.e. first order) functions. Accordingly, under Model 2, with parental age effects fitted as linear covariates we estimated highly significant negative effects of maternal and paternal age on offspring speed that were of similar magnitude (table 1). From this model, the estimated effect (s.e.) of paternal age (−0.006 (0.0004) yards s^−1^ yr^−1^) was actually slightly more negative than that of maternal age (−0.005 (0.0003) yards s^−1^ yr^−1^). We do not formally compare these estimates but note approximate 95% CI (calculated as coefficient ± 1.96 s.e.) are strongly overlapping and no meaningful difference is inferred.

**Table 1.** Estimated effects of parental age on offspring speed from Models 2–4 in which linear effects of parental age are modelled. In Model 2, simple linear effects of maternal and paternal age at offspring conception are fitted. In Model 3, each of these is decomposed into an effect of mean parent age (across offspring within each parent) and a within-parent deviation (Δ) from that (see main text). This yields estimates of within-parent age effects that are expected to be robust to any selective disappearance (or appearance) in the dataset. In Model 4, effects of mean parent age (across offspring within each parent) are again estimated, but this time conditional on actual parent ages for each offspring observation. These can be interpreted as estimates of selective disappearance. Statistical inference is by conditional Wald *F*-tests implemented in ASReml.

effect	coefficient (s.e.)	*F*	d.f.	*p*	interpretation
Model 2					
maternal age	−0.005 (0.0003)	284.39	173 087.0	<0.001	maternal age effect subject to selective disappearance bias
paternal age	−0.006 (0.0004)	202.89	13 595.7	<0.001	paternal age effect subject to selective disappearance bias
Model 3					
$\bar{\mathrm{maternal}\;\mathrm{ag}e_{j}}$	0.003 (0.0004)	84.94	139 532.7	<0.001	
$\;\mathrm{dam}\Delta_{ij}$	−0.017 (0.0004)	1385.45	173 686.0	<0.001	within-dam age effect robust to selective disappearance
$\bar{\mathrm{paternal}\;\mathrm{ag}e_{k}}$	0.006 (0.0009)	43.43	11 674.0	<0.001	
$\;\mathrm{sire}\Delta_{ik}$	−0.011 (0.0006)	389.73	14 537.5	<0.001	within-sire age effect robust to selective disappearance
Model 4					
maternal age	−0.017 (0.0004)	1382.79	173 667.5	<0.001	
paternal age	−0.011 (0.0006)	390.66	14 540.9	<0.001	
$\bar{\mathrm{maternal}\;\mathrm{ag}e_{j}}$	0.020 (0.0006)	1182.96	167 981.5	<0.001	selective disappearance of dams
$\bar{\mathrm{paternal}\;\mathrm{ag}e_{k}}$	0.017 (0.0012)	209.24	12 084.0	<0.001	selective disappearance of sires

**Figure 2.**
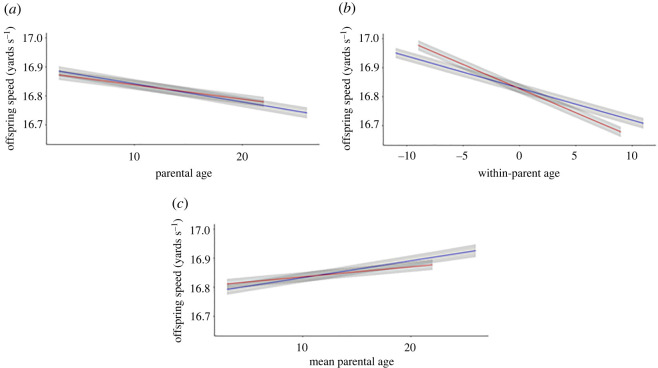
Predicted linear effects of maternal and paternal age on offspring speed showing (*a*) partial linear regressions of maternal (red) and paternal (blue) age on offspring speed estimated under Model 2. Since these estimated age effects are potentially biased by selective disappearance effects, they were then decomposed into (*b*) effect of within-parent ageing and (*c*) effects of mean parent age using Model 3. Differences in slopes between within-parent ageing (*b*) and mean parent age effects (*c*) is explained by selective (dis)appearance of dams and sires. Shaded areas represent ± 1 s.e.

Models 1 and 2 thus show a clear pattern of decline in average offspring speed with increasing parental age at the population level. Model 3, in which we used mean-centring, reveals these patterns at the population level are driven by significant within-parent effects. Under Model 3, our estimates of within-sire and within-dam age effects (s.e.) were −0.011 (0.0006) and −0.017 (0.0004) yards s^−1^ yr^−1^, respectively (table 1). Accepting approximate 95% CI as equalling the coefficient ± 1.96 s.e., and noting that non-overlapping CI implies a significant difference at *α* = 0.05, two points arise. First under Model 3, both of these effects are considerably, and significantly, more negative than the corresponding unpartitioned parental age effects from Model 2 (see above; figure 2). Second, based on Model 3, the negative impact on offspring speed of within-parent increase in age is (significantly) stronger for dams than sires (figure 2). Furthermore, differences between the negative within-parental ageing slopes and positive mean parent slopes (i.e. between the coefficients for $\mathrm{dam}\Delta_{ij}$ and $\bar{\mathrm{maternal}\;\mathrm{ag}e_{j}}$ in the maternal case) suggest selective (dis)appearance is occurring in both dams and sires. Model 4 provides statistical support for this inference as significant differences for both dams and sires were found (table 1).

Random effects included were all statistically significant. Using the Model 3, fixed effect specification, likelihood ratio test comparisons to reduced models confirmed the presence of significant variation in speed among-offspring (within-parents; $\chi_{0,1}^{2}=36948.0,$ *p* = <0.001), among-dams ($\chi_{0,1}^{2}=645.6,$ *p* = <0.001) and among-sires ($\chi_{0,1}^{2}=4096.5,$ *p* = <0.001). Scaled to intra-class correlations (s.e.) these effects explain 18.9 (0.17), 2.7 (0.12) and 4.3 (0.23) % of variation in speed (conditional on fixed effects), respectively. Note that our random effect structure is formulated to prevent pseudoreplication of fixed effects, and we caution that these variance components do not have simple causal interpretation. Offspring, dam and sire identity variances will contain an unknown mix of genetic and environmental effects (with *V*_dam_ and *V*_sire_ also including repeatable parental identity effects if present).

## 4. Discussion

We set out to test for, and estimate the magnitude of, parental age effects on offspring racing speed in the thoroughbred racehorse population. To achieve this, we used a larger, more comprehensive dataset than any previous study which included records of 906 027 performances by 101 257 offspring runners produced from 41 107 dams and 2887 sires. Our analysis identified significant negative within-parent effects of advancing maternal and paternal age on offspring speed. While we had expected to find some evidence of parental age effects, their magnitude is notable, particularly that of the previously undocumented influence of paternal age on offspring speed. Thus, our study adds to the small but growing literature on parental age effects on commercially important traits in livestock and shows that senescent declines in parental performance occur in both sexes of thoroughbred racehorses.

Using the within-individual centring approach to prevent bias from selective disappearance, we estimated a within-dam age effect on offspring speed of −0.017 (0.0004) yards s^−1^ yr^−1^. Qualitatively, this finding recapitulates the pattern previously reported for maternal age effects [37–39]. Quantitative comparison to these earlier papers is difficult as they analysed Timeform ratings [37] and degree of success in races [38,39] as opposed to speed itself as a measure of offspring performance. These studies were also much smaller than the present analysis and potentially yielded results biased by selective disappearance (discussed more below). For context we note that our predicted effect size translates into a predicted difference of approximately 1 s over a race of 1760 yards (1 mile) between, by way of example, a 5-year-old and a 15-year-old mother. This may seem a small effect size, but in fact the average winning margin of 1760 yard races in our dataset was just 0.3 s.

Perhaps more surprising was that we also documented a large within-sire paternal age effect. The effect size is less than that for the maternal age effect, but of the same order of magnitude (at −0.011 (0.0006) yards s^−1^ yr^−1^ or 65% of the maternal estimate). We are not aware of any previously published studies that unequivocally demonstrate (within-sire) paternal age effects on racehorse performance. However, that such effects are possible has long been recognized [40] and highlighted within the industry in relation to performance of offspring from leading sires (i.e. producers of high-performing offspring [41,42]) despite the expectation that these valuable stallions will be mated to the best mares and have offspring trained at leading establishments. These previous industry reports analysed repeated measures on a non-representative group of ‘leading’ sires and may have been vulnerable to bias caused by ‘regression toward the mean’ [50]. Specifically, if high sire quality is erroneously inferred from strong early life performances by a truly average sire, then increasing age brings more data and the estimated merit of that sire will decline towards the mean. In this study, we avoid this potential source of bias by analysing all sires represented irrespective of perceived (or actual) quality.

Here we used within-individual centring to obtain putatively unbiased estimates of within-parent age effects on offspring speed. Under Model 3, the gradient of within-parent effects of advancing age were significantly negative (for both maternal and paternal age), while the mean parental age effects were significantly positive (for both dams and sires), implying selective (dis)appearance of both dams and sires from our dataset with advancing parental age. The latter was confirmed by Model 4, in which selective (dis)appearance of both dams and sires was found to be significant. Given the competitive nature of the thoroughbred breeding industry in Great Britain, the most parsimonious explanation for this is that sires and dams are removed from the breeding population at earlier ages if their offspring fail to prove successful. In fact, in addition to selective disappearance of parents with unsuccessful offspring, there may also be some selective appearance of high-quality parents at later breeding ages (e.g. if parents that have proven successful elsewhere are imported into the thoroughbred breeding population of Great Britain). However, the distinction is of limited value for current purposes since both selective processes have the same consequence; better than average parents will have higher than average ages at reproduction in the data. Despite the fact that selective disappearance can mask the signature of within-individual senescence [44], significant negative effects of parental age were still estimated under Model 2. Thus, in this case, failure to account for selective (dis)appearance leads to underestimation of parental senescence for offspring speed, but the bias is not sufficient to completely mask it.

The mechanisms underpinning the parental age effects detected here are unknown. Intuitively, maternal age effects may be mediated through declining quality of parental care. As well as primiparous mares, older multiparous females have been found to produce lighter foals [36,51], and lighter offspring are viewed less favourably within the industry [34,52]. However, in a cohort study of 409 UK-born foals, no association between foal weight and later race performance was detected [53]. This followed an earlier and larger study of 3734 foals in the USA, in which neither foal weight nor wither height predicted performance [54]. Thus, there is evidence that offspring weight declines at later maternal ages in thoroughbreds, a finding that is common across captive and wild populations of mammals more generally [55]. However, there is currently no robust evidence that the decline in offspring speed with maternal age is mediated by effects on offspring size. Besides, older mothers can influence offspring independent of maternal care and offspring weight [21,56].

Moreover, since sires provide no care to offspring in commercial thoroughbred breeding, senescent declines in parental care cannot be a generally sufficient explanation for the effects detected. Epigenetic mechanisms are certainly plausible, can be age-sensitive [14,21,57] and can sometimes be transmitted across multiple generations [58,59]. This raises the question of whether parental age effects as detected here could even cascade across further generations (e.g. giving rise to grandparental age effects). There has been speculation that leading sires might themselves be offspring of younger mares [41]. Other plausible candidate mechanisms may include accumulation of germline DNA mutations with age [18,60–62], and/or interactions of epigenetic signalling pathways with telomere length dynamics [18].

A final point to note is that while we have modelled additive effects of within-mother and within-father ageing, there could sometimes be mechanisms by which parental age effects combine multiplicatively (as tested for but not detected in [29]). However, in a *post hoc* analysis suggested at review, we found no support for this. Specifically extending Model 3 to include the interaction of within-parent ageing (damΔ:sireΔ)  yielded an estimated effect size that was small and not statistically significant (coefficient (s.e.) = −0.00016 (0.00014), *p* = 0.243; full results not shown).

## 5. Conclusion

In summary, here we report evidence that increasing maternal and paternal age negatively impacts racehorse speed. Although maternal age effects were stronger (more negative), the existence and magnitude of paternal age effects is particularly noteworthy as it has not been recognized previously. Our results suggest that selective (dis)appearance of both sires and dams may, to some extent, have contributed to a failure to realize the magnitude of within-parental declines over age. These findings, for a trait of commercial importance, could potentially be used to optimize breeding decisions with respect to both target traits (offspring speed) and financial inputs (e.g. stud fees) within the thoroughbred industry. Although the present study is clearly not informative for mechanism, we also hope that our findings will stimulate research into the pathways by which parental age influences are transmitted to offspring phenotype.

## Data Availability

Supporting data and code are archived in Dryad https://doi.org/10.5061/dryad.qbzkh18m0 [63]. The data are provided in the electronic supplementary material [64].
